# The impact of visual fidelity on screen-based virtual reality food choices: A randomized pilot study

**DOI:** 10.1371/journal.pone.0312772

**Published:** 2025-01-30

**Authors:** Bianca Curi Braga, Pejman Sajjadi, Mahda Bagher, Alexander Klippel, Jessica Menold, Travis Masterson

**Affiliations:** 1 Department of Nutrition, College of Human Health and Development, The Pennsylvania State University, University Park, PA, United States of America; 2 College of Computing and Software Engineering, Kennesaw State University, Kennesaw, GA, United States of America; 3 Meta, Redmond, WA, United States of America; 4 Department of Environmental Sciences, Wageningen University & Research, Wageningen, Netherlands; 5 Department of Mechanical Engineering, College of Engineering, The Pennsylvania State University, University Park, PA, United States of America; University of Marburg: Philipps-Universitat Marburg, GERMANY

## Abstract

**Objective:**

To understand the impact of fidelity and perceived realism on virtual reality food choices, and task motivation, engagement, and interest.

**Design:**

Randomized controlled trial.

**Setting:**

Online.

**Participants:**

84 participants recruited from Amazon Mechanical Turk.

**Intervention:**

Participants were randomly assigned to either a high- (n = 43) or a low- (n = 41) visual fidelity environment and were asked to select foods to have a meal with a friend.

**Main outcome measures:**

Food choice, motivation of food choices, engagement, and interest.

**Analysis:**

Simple linear regressions between visual fidelity and perceived realism, and log-linear regressions for visual fidelity or perceived realism on either motivation, interest, or engagement. Poisson models between visual fidelity or perceived realism, and food selections.

**Results:**

Manipulating visual fidelity was not associated with perceived realism, motivation, interest, or engagement in the food selection task. Perceived realism increased motivation by 0.3% (SE 0.056; p = 0.022), interest by 1.4% (SE 0.002; p<0.001), and engagement by 0.9% (SE 0.001; p<0.001) in the food selection task. High visual fidelity decreased the total number of foods selected (B = 0.216; CI (-0.384; -0.047); p = 0.012).

**Conclusion and implications:**

Perceived realism, but not visual fidelity, is important for task related factors like motivation, engagement, and interest. Visual fidelity may influence some food selections.

## Introduction

The selection of foods for consumption relies on both intrinsic (i.e., individual motivations) and extrinsic (i.e., environmental contexts) factors [1–6]. Consumers often must balance the rewarding properties of a food against a variety of perceived short and long-term consequences of consuming them [7]. For example, it has been demonstrated that food selection is based on costs, sensory quality (e.g., taste, texture, smell), convenience, nutrition value, environmental impact, health benefits, and social issues [8–10].

When investigating food choice, most studies rely on hypothetical scenarios and often employ visual representations of foods. Novel technologies such as virtual reality (VR) have the potential to enhance our comprehension of food choices and the motivations driving them. Food selection in a VR food buffet, for example, have been shown to reflect food selection made in real food buffets [11–13]. VR makes the gap between the virtual and the real experience smaller by providing ecologically valid scenarios that reflect many aspects of real-life experiences. VR and real-life experiences can be perceived similarly [14]; but perception of realism dependent on the illusion of presence (degree of technical immersion) and the plausibility of the hypothetical situation (degree of credibility of scenario) [15].

Through experiences such as presence and embodiment, VR has the potential to afford users natural and realistic means of interaction with foods and environments [16]. Visual fidelity, or the extent to which an image looks like a photo rather than generated by a computer [17], may influence users’ perception of virtual foods, their engagement in virtual experiences, and virtual food choices [18]. For example, a recent study demonstrated that the visual quality of digital food items can influence participants desire to consume them [19].

Visual fidelity depends on geometric realism (how much virtual objects seem like their real-world counterparts) and illumination realism (lighting fidelity) [20]. In relation to digital imagery, high visual fidelity means realistic, while low visual fidelity is simpler and more cartoonish [21]. High visual fidelity can potentially make VR-based scientific experiments more realistic and engaging [21], but the extent to which manipulating visual fidelity predicts user experience and behavior in relation to virtual food selection is unclear. To the best of our knowledge, only one study has systematically manipulated the visual fidelity of foods [19] although they did not manipulate the visual fidelity of the surrounding environment where food was displayed in the simulation.

Therefore, this pilot study sought to understand if manipulating visual fidelity affects users’ perception of task realism and if either visual fidelity or perception of realism are related to motivation, interest and enjoyment, or engagement within a food selection task. Our goal was to test the effect of different levels of visual fidelity and the perception of realism of the virtual foods and environment where they were displayed on virtual food choices. To accomplish this, we created two versions of a virtual food selection task that differed in visual fidelity. Difference in visual fidelity of foods and the environment were achieved by manipulating lighting, geometry [22], rendering quality [23], and texture quality [24] of food items and the surrounding environment. In this study we hypothesized that higher visual fidelity would be associated with higher levels of perceived realism and increased motivation, interest and engagement in the food choice task. We also hypothesized that perceived realism, regardless of visual fidelity, would be related to motivation, engagement, and interest in the food choice task. Finally, we hypothesized that interacting with high fidelity models leads to a larger number of foods being selected.

This is an important topic of study, given the costs and effort for producing high visual fidelity models. If low visual fidelity has similar or better user performance to high visual fidelity, it will help researchers save money in application development and produce training content more quickly [21]. This is particularly pertinent to academia where both financial and human resources are scarce.

## Materials and methods

In this study we measured the impact of visual fidelity and perceived realism on food choice, food choice motivation, interest, and engagement by randomly assigning 88 participants to either a high or a low visual fidelity condition on a web-based VR experience. Participants were recruited from Amazon Mechanical Turk (MTurk). Remote studies ran on MTurk have shown to be an effective way in obtaining a heterogenous sample with adequate diversity of participants, capturing reliable data, and saving costs [29–32]. Our excluding criteria were (1) repeating the survey, (2) leaving the surveys incomplete, (3) spending 2.2 standard deviations from the mean time to finish the study [25]. No one was excluded based on the first two criteria, but four participants were excluded for taking too long to complete the study. We analyzed the data from 84 participants, 43 in the high visual fidelity and 41 in the low visual fidelity conditions. The Human Research Protection Program Institutional Review Board of The Pennsylvania State University determined that this study (#00019284) did not require formal IRB review because it met the criteria for exemption according to the policies of The Pennsylvania State University and the federal regulations. The study started on January and ended in June of 2022. The participants provided informed written consent. We followed the principles of the Declaration of Helsinki. The data collected are available in the S1 Data.

### Intervention

Two versions of the same virtual experience were created, one with high visual fidelity models and one with low visual fidelity models. The virtual experience represented a kitchen with 21 different food items grouped as fruits & vegetables, main course or dessert, and placed on a table at the center of the scene. The users could change the angle of the camera view (rotate) and zoom in or out to explore and examine the food items from different perspectives. The virtual experience was developed in Unity3D and was made accessible through conventional web browsers.

### Manipulating fidelity

**Fig 1:**
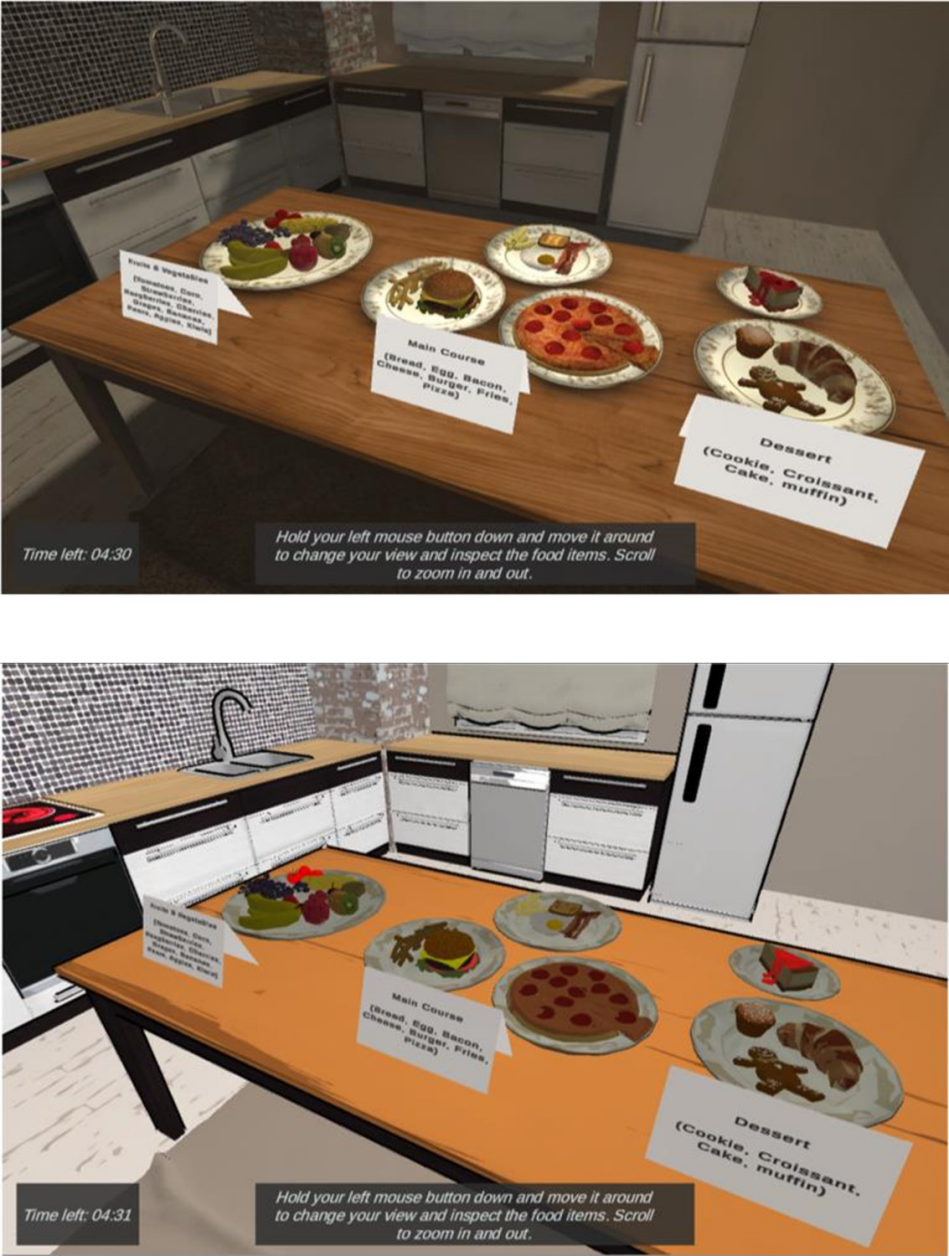
High and low fidelity scenes. The top image shows the high-visual fidelity and bottom shows the low-visual fidelity condition.

**Fig 2:**
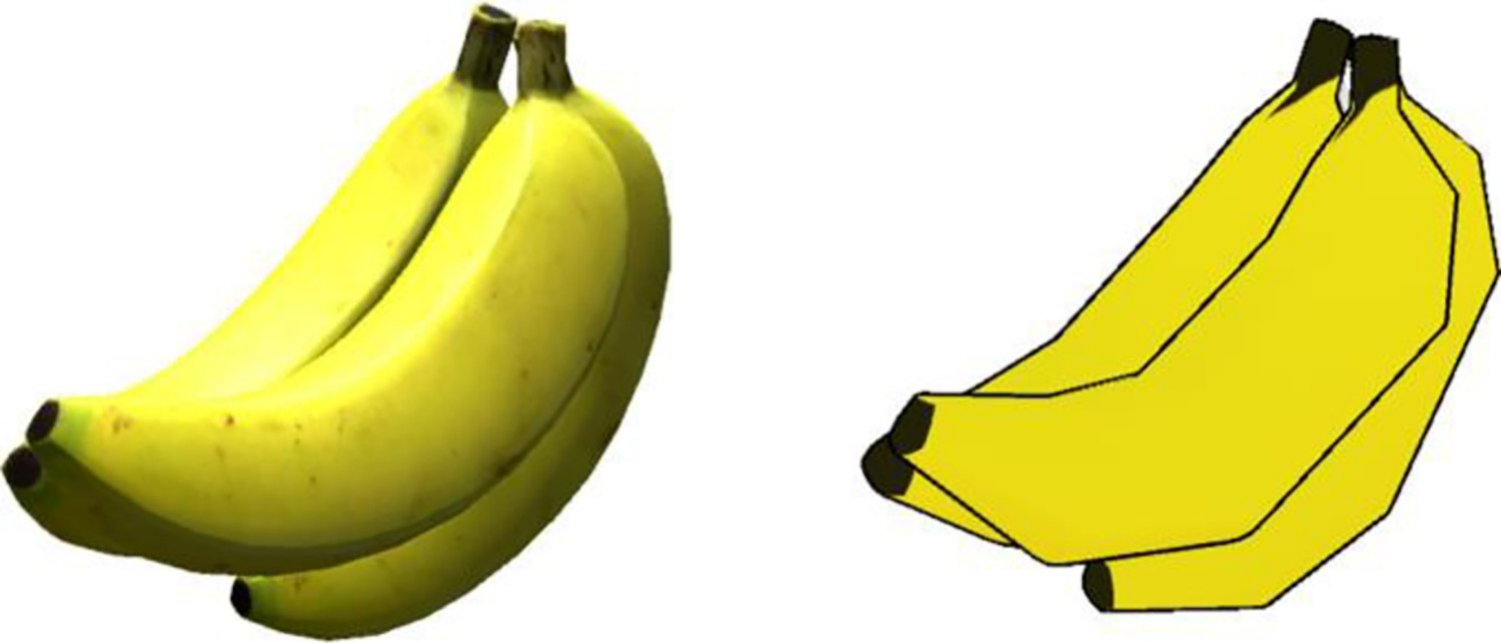
Example of differences in visual fidelity of the food items. The left image shows the high-visual banana and the right image shows the low-visual fidelity banana.

The visual fidelity of the virtual experience was manipulated through a process in which we purchased high fidelity photogrammetry models, and systematically reduced their visual quality through removal of details and stylization. A specific collection of models with a wide variety of high-quality food objects based on real photographs was used [26]. It was our aim to maintain a good level of similarity between the two levels of visual fidelity in terms of recognizability. As such, we utilized a top-down approach where we removed detail from the base high-fidelity models to synthesize low fidelity ones. This process included reducing polygon counts and modifying textures. Polygon reduction was automatically processed, while reducing texture quality involved applying the cutout filter in Adobe Photoshop [27]. Furthermore, a toon shader was applied to the synthesized lower fidelity models to intensify the reduction effect The toon shader gave the “flat” look to the objects in the low visual fidelity condition. Toon shaders also include an outline around the object to make it stand out distinctly. Toon shaders have been applied previously to give cartoonish appearance to 3D characters in a medical virtual reality program [28]. Research has shown that intentionally making images more cartoonish avoids the uncanny valley, or the aversion to almost but not quite realistic avatars, for example [29]. When the toon shader we used was paired with the polygon and texture reduction, the result was a good balance of reduced quality while retaining enough fidelity to enable easy recognition. Colors of the foods were not manipulated beyond this procedure. **Fig 1** shows the high and low visual fidelity environments. **Fig 2** shows the difference between a high- and low-visual fidelity banana (polygon counts of 4860 and 940, respectively).

### Study procedure

The study began with the participants providing consent and answering a set of pre-stimuli questions. Upon completion, they received a code to start the VR experience and then another code to continue with the post-stimuli questions once the VR experience was over. The instructions explained that they should explore the 21 food items on the table of the virtual kitchen and make food choices to have a meal with a friend. They explored the virtual foods for three to five minutes from different angles by rotating around and zooming in and out using a mouse. After three minutes, they were allowed to end the VR experience and start the post-stimuli questionnaire. If they reached the end of the five-minute threshold, the experience would automatically end. In the post-stimuli survey, participants selected foods for a hypothetical meal with a friend and answered questions about the VR experience. After finishing the study, they received a code to enter into MTurk to be compensated for participation.

### Pre- and post-VR surveys

All questions from the pre- and post-VR surveys had either Likert scale or multiple-choice answers or asked to enter a value. Appendix 1 shows the pre- and post-VR surveys. We used the Eating Motivation Survey (TEMS), that encompasses a broad spectrum of motives for normal eating behavior (i.e., food choice) in a validated, psychometric-based, comprehensive systematic survey [30]. Interest and enjoyment (herein referred as interest) were measured using five questions from the modified version of the interest-enjoyment subscale [31]. Engagement was measured using three questions from a 32-question scale related to realism, sensory fidelity, and distraction factors [32]. We measured individuals’ external food cue reactivity as a measure of eating behavior using an adapted version of the scale developed by [33]. Fullness rating was measured pre-survey using a visual analogue scale (VAS) to control for the hunger level of participants as a potential confound. We measured perceived realism of the virtual experience in light of the perception of typicality, plausibility, and perceptual quality. Typicality, plausibility, and perceptual quality measured how typical, plausible and realistic participants thought the virtual environment and scenario were. We measured perceived realism as part of the post-VR survey using an adapted version of the scale proposed by [18].

### Data analysis

To investigate the relationship between visual fidelity and perceived realism we ran a log-linear regression with visual fidelity as the independent variable and perceived realism as the dependent variable. We followed this with a sensitivity analysis correcting for participants fullness ratings, external food cue reactivity ratings, and ethnicity. We also ran log-linear regressions using visual fidelity and perceived realism as independent variables and either task motivation, interest, or engagement as the dependent variables. In all models we used a logarithmic transformation to make the betas interpretable since food choice motivation, interest, and engagement were the sums of Likert scale scores from each individual survey.

To investigate the relationship between visual fidelity, perceived realism, and food selections we ran separate Poisson regressions for visual fidelity and perceived realism as the independent variable on foods chosen including the number of low-energy dense foods, the number of high-energy dense foods, and the total number of overall foods chosen. We also ran a combined model that included both visual fidelity and perceived realism in the same model while also correcting for external food cue reactivity, fullness rating, and ethnicity. We conducted similar follow up analyses using linear regressions with estimated kilocalories (kcal) of foods selected rather than the number of foods. We also stratified this by low-energy dense foods, high-energy dense foods, and total foods. As an exploratory analysis we analyzed how differences in visual fidelity and perceived realism affected the selection of any of the 21 individual foods displayed. To do this we used Probit regressions. We again used a follow up analysis examining estimated kcal of foods selected using linear regression. The significance level for all tests conducted was established *a priori* as a p<0.05.

## Results

### Participant demographics

The average age of our sample was 39.8 (11.9) years. White/Caucasians were 75% and black were 16% of the analyzed participants. Half of the sample were female, 39% were college educated or more, and 20% had an income lower than the median income of the United States [34]. High- and low-visual fidelity groups were balanced at baseline for all characteristics but being Hispanic or Latino (p = 0.053). Participant groups differed in fullness rating at baseline (p = 0.034). The demographics are described in **Table 1**.

**Table 1:** Participants’ characteristics at baseline.

	Low visual fidelity	High visual fidelity	T-test
Age mean in years (SD)	37.86 (11.86)	41.78 (9.37)	0.098
Race			
American Indian/Alaskan	1 (2.33)	0 (0.00)	0.332
Asian	4 (9.30)	1 (2.44)	0.188
Black/African American	8 (18.60)	5 (12.20)	0.423
White/Caucasian	29 (67.44)	34 (82.93)	0.104
Mixed/other	1 (2.33)	1 (2.44)	0.973
Ethnicity			
Hispanic/Latino	8 (18.60)	2 (4.88)	0.053*
Female biological sex	22 (51.16)	20 (48.78)	0.830
Female gender	22 (51.16)	20 (48.78)	0.830
College degree or more	17 (39.53)	16 (39.02)	0.962
Income <69,000/year	9 (20.93)	8 (19.51)	0.873
	n = 43	n = 41	* *

Answers are in number of participants (percentage of participants in the condition), but age in years (standard deviation).

### Relationships between visual fidelity, perceived realism, and motivation, interest, and engagement

The log-linear regression between visual fidelity and perceived realism showed that there was no significant relationship between visual fidelity and perceived realism (B = 0.04, SE = 0.07, p = 0.570). Log-linear regression models are a type of generalized linear model that are used for modeling categorical data. The model uses a log-link function to relate the linear predictors to the response variable. In other words, it’s possible to interpret the impact of the independent variables in the dependent variable as a percentage. The results of the log-linear regression models investigating the relationship between visual fidelity, perceived realism, motivation, interest, and engagement are summarized in **Table 2**. In short, visual fidelity was not directly associated with motivation, interest, or engagement in the food selection task. This was true even in the combined model and when controlling for external food cue reactivity, fullness rating, and ethnicity. However, perceived realism was associated with all three independent variables. Specifically, our models showed that a one-point change in perceived realism independently increased overall motivation in the task by 0.3% (SE 0.05; p = 0.022), interest in the task by 1.4% (SE 0.002; p<0.001), and engagement 0.9% (SE 0.001; p<0.001) in the food selection task. As perceived realism could range from 15 to 105 points, a 30 points difference between subjects’ perception of realism, for example, would suggest a 9% difference in motivation between the two participants. This was true even in our combined models which controlled for external food cue reactivity, fulness rating, and ethnicity.

**Table 2:** Log-linear regression results for motivation, interest, engagement, and perceived realism.

	Motivation				Interest				Engagement			
	B	SE	*p*	*n*	B	SE	*p*	*n*	B	SE	*p*	*n*
**Simple Models**												
Visual fidelity	-0.046	0.06	0.42	73	-0.016	0.09	0.85	84	0.04	0.06	0.52	84
Perceived realism	0.003	0.00	0.007*	72	0.014	0.00	<0.001*	83	0.009	0.00	<0.001*	83
**Combined Corrected Models** ^**a**^												
Visual fidelity	-0.016	0.06	0.77	71	-0.059	0.07	0.41	81	0.03	0.05	0.52	81
Perceived realism	0.003	0.06	0.02*		0.014	0.00	<0.001*		0.009	0.00	<0.001*	
External food cue reactivity	0.018	0.01	0.004*		0.015	0.01	0.06		0.006	0.01	0.27	
Fullness rating	0.009	0.01	0.09		-0.005	0.01	0.50		0.001	0.00	0.75	
Ethnicity	0.058	0.09	0.51		-0.098	0.11	0.39		0.002	0.07	0.98	

SE is standard error. Significance based on 95% CI. Separate log-linear regressions were run for high visual fidelity and perceived realism.

^a^Log-linear regression with high visual fidelity adjusted for perceived realism, fullness rating, external cue reactivity, and ethnicity. Visual fidelity: dummy variable with high = 1 and low = 0.

### Relationships between visual fidelity, perceived realism, and food choice

The results of the Poisson regressions for visual fidelity and perceived realism as independent variables on food choice using the total number of foods chosen are summarized in **Table 3**. The main difference between a Poisson regression and a log-linear regression is that the Poisson regression is used when the primary goal is to model count data where the dependent variable represents the count of occurrences of an event. Perceived realism was not associated with any of the food selection variables. However, visual fidelity was associated with food selections with significantly less foods being selected in the high-fidelity condition (B = 0.216; CI (-0.38; -0.05); p = 0.012). This was driven by the number of high energy dense foods selected (B = 0.322 (CI (-0.55; -0.09); p = 0.006) in the high-fidelity condition but not the number of low-energy foods. These results remained significant even in our combined model when controlling for perceived realism, external food cue reactivity, fullness rating and ethnicity. The results of the linear regressions, when food choice was modeled as the sum of estimated kilocalories (kcal) rather than just number of foods, are summarized in **Table 4**. These regressions also showed an effect of visual fidelity for all foods (B = -385.49; CI (-688.02; -82.97); p = 0.013) and for high energy dense foods (B = -400.10; CI (-712.47; -87.72); p = 0.013). Food selection results remained consistent even when controlling for perceived realism, external food cue reactivity, fullness rating and ethnicity.

**Table 3:** Poisson regressions for the total number of low-energy dense foods, of high calorie density foods and the sum of all foods.

	Sum number low-energy dense foods				Sum number high-energy dense foods				Sum number all foods			
	B	95% CI	*p*	n	B	95% CI	*p*	n	B	95% CI	*p*	n
**Simple models**												
Visual fidelity	-0.089	(-0.34; 0.16)	0.48	84	-0.322	(-0.55; -0.09)	0.006*	84	-0.216	(-0.38; -0.05)	0.012*	84
Perceived realism	0.006	(-0.00; 0.01)	0.10	83	-0.004	(-0.01; 0.00)	0.13	83	0.000	(-0.00; 0.00)	0.99	83
**Combined corrected models** ^**a**^												
Visual fidelity	-0.004	(-0.28; 0.27)	0.98	81	-0.331	(-0.58; -0.08)	0.009*	81	-0.183	(-0.37; -0.00)	0.05*	81
Perceived realism	0.005	(-0.00; 0.01)	0.16		-0.004	(-0.01; 0.00)	0.21		0.000	(-0.01; 0.01)	0.99	
External food cue reactivity	0.011	(-0.02; 0.04)	0.47		0.008	(-0.02; 0.04)	0.58		0.009	(-0.01; 0.03)	0.38	
Fullness rating	0.010	(-0.02; 0.04)	0.42		0.004	(-0.02; 0.03)	0.76		0.006	(-0.01; 0.02)	0.45	
Ethnicity	0.147	(-0.24; 0.54)	0.46		-0.201	(-0.59; 0.19)	0.31		-0.041	(-0.32; 0.23)	0.77	

Significance based on 95% CI. ^a^ Poisson regression adjusted for perceived realism, external food cue reactivity, fullness rating, and ethnicity. Visual fidelity was a dummy variable with high = 1 and low = 0.

**Table 4:** Linear regressions for the kcal sum of low-energy foods, of high energy dense foods and for all foods.

	Sum kcal of low-energy dense foods				Sum kcal of high-energy dense foods				Sum kcal of all foods			
	B	95% CI	*p*	n	B	95% CI	*p*	N	B	95% CI	*p*	n
**Simple models**												
Visual fidelity	- 14.604	(-76.03; 46.82)	0.64	84	-385.494	(-688.02; -82.97)	0.013*	84	-400.098	(-712.47; -87.72)	0.013*	84
Perceived realism	1.307	(-0.31; 2.92)	0.11	83	-5.483	(-13.79; 2.87)	0.19	83	-4.176	(-12.80; 4.49)	0.34	83
**Combined corrected models** ^**a**^												
Visual fidelity	2.861	(-64.15; 69.87)	0.93	81	-403.124	(-738.69; -67.55)	0.019*	81	-400.263	(-749.31; -51.21)	0.03*	81
Perceived realism	1.184	(-0.49; 2.86)	0.16		-4.768	(-13.14; 3.60)	0.26		-3.584	(-12.29; 5.12)	0.46	
External food cue reactivity	2.499	(-4.81; 9.81)	0.50		14.024	(-22.59; 50.64)	0.45		16.523	(-21.56; 54.61)	0.39	
Fullness rating	3.325	(-3.07; 9.72)	0.30		0.730	(-31.30; 32.76)	0.96		4.054	(-29.26; 37.37)	0.81	
Ethnicity	16.508	(-88.31; 121.33)	0.76		-279.635	(-804.55; 245.28)	0.29	** **	-263.127	(176.66; 2682.38)	0.34	** **

Significance based on 95% CI. ^a^ Linear regression controlled by perceived realism, external food cue reactivity, fullness rating, and ethnicity. Visual fidelity was a dummy variable with high = 1 and low = 0.

Results of fidelity when considering the selection of each individual food item are summarized in **S1** and **S2 Tables.** In short, four food items were selected significantly more in the low- than in the high-visual fidelity condition: eggs (B = -0.745; SE = 0.29; p = 0.01); bacon (B = -0.533; SE = 0.30; p = 0.05); cheese (B = -0.600; SE = 0.31;

p = 0.05); croissant (B = -1.000; SE = 0.35; p = 0.005). Perceived realism was associated with choosing more corn (B = 0.014; SE = 0.01; p = 0.10); more pears (B = 0.036; SE = 0.01; p = 0.006) and less eggs (B = -0.015; SE = 0.01; p = 0.05). Only the significance of pear (B = 0.035; SE = 0.01; p = 0.012), eggs (B = -0.016; SE = 0.01; p = 0.05) and bacon (B = 0.013; SE = 0.01; p = 0.10) were maintained in the combined models, that adjusted visual fidelity by perceived realism, fullness rating, external food cue reactivity, and ethnicity.

## Discussion

As described previously, VR models can exhibit different degrees of visual fidelity, which may influence VR-based study outcomes. This study tested the impact of high compared to low visual fidelity environment and objects and how this may modify perceived realism and relate to task related motivation, interest, and engagement during a food selection task in a web VR environment. Our findings suggest that visual fidelity was not associated with motivation, interest, and engagement, nor with perceived realism. In contrast, perceived realism was associated with motivation, interest, and engagement. This suggests that the perception of realism by users does not necessarily depend on the technological visual fidelity of the experience, but perhaps does more on the experiential typicality and plausibility it elicits in users. This observation has rather important implications for the design and operationalization of virtual experiences, particularly with serious applications. The perceived realism of an experience is shown to be a determining factor in users’ motivation, interest, and engagement, and should therefore be a key indicator of a good user experience. Nevertheless, when designing VR experiences, developers should focus on the plausibility and typicality of the experience rather than the merely the visual fidelity of it.

While visual fidelity was not related to task engagement measure it did appear to be weakly related to food choice. This appears to partially support previous work [19] in which higher visual fidelity of a food stimuli was related to a higher desire to eat that food. The response to visual fidelity in their study reflected a minimum required quality to trigger a significant desire to eat. More specifically, going from level one to two within a one (low) to seven (high) scale of visual fidelity led to no effect on desire to eat, but the increase in visual fidelity led to higher desire to eat from level two and on, with diminishing returns at top levels [19]. Our work suggests that some but not all foods may be impacted by changes in visual quality, however future studies are required to tease apart why this may be the case.

In contrast with [19], our study manipulated both foods and the surrounding virtual environment. Additionally, our study was designed to understand the effect of the complete context, considering both the foods presented and the virtual kitchen where it was displayed. We did this because both food and the environmental context can influence food behavior and desire to eat [19]. For example, the context in which food is experienced can alter its appeal [35]. Altering both the foods and the environment may be important for the congruity of the virtual experience [35]. Moreover, we rarely consume foods isolated from the environment in natural experiences, such as within sensory booths [35]. This design, however, limited our ability to parse the effects of the environment versus the effect of the food as a focal object. We could not reconcile the effect of the kitchen’s fidelity on the sense of presence of the participant, which could influence the evaluation of the foods themselves. We plan to address this problem in a future study through placing the high and low fidelity foods in different types of environments and see if that influences the reactions of participants. For example, we can place the high and low fidelity foods in a 3D environment vs an environment with a photo as backdrop vs a gray-box environment as control.

Overall, our findings show that perception of realism, that is, the believability of the visual stimuli and the task, but not the visual fidelity at which some foods are modeled, could be an important consideration. These results have implications for specific study designs and for testing in specific populations. Specifically, it may be important to assess and control for perceived realism within study designs. Additionally, if a study relies on the selection of specific foods, it may be important to pilot the stimuli prior to use within a virtual experiment to assess its fidelity and perception to ensure that the food model itself is not impacting study results. It is also important to consider that other studies have shown that visual fidelity can alter the effect of training paradigms [36]. However, this should be balanced against constraints such as processing power of available equipment and the effort, cost, and time needed to model objects precisely. Our data suggest that the perception of visual fidelity, rather than hyper realistic modeling, may be more important. Therefore, VR may be able to provide more ecologically valid conditions for learning and performing tasks [13, 32, 37, 37–41] without the need for hyper realistic food models. However, our findings are preliminary and future work is needed to understand what aspects (i.e., lighting, context, colors) improve the perception of lower-visual fidelity models. This would lead to effective model designs that could be used with a variety of technologies at a lower cost.

### Strengths and limitations

A strength of remote experiments like this one is that they offer a practical solution to simulate realistic and validated interactions with food at scale, while having more nuanced results at lower cost and no food waste [25, 42, 43]. Most experiments on food selection for commensality and most human-computer interaction studies have been conducted in laboratory (lab) settings [44–48]. Lab studies have homogeneous participants, usually college students, leading to limited external validity and contradictory findings in the literature. In-person VR studies require a dedicated open space in the lab, besides the burden of scheduling and managing participant’s recruitment [49–52].

Our sample had about the gender/sex and race proportion of the general American population [53]. The groups were not balanced in terms of fullness rating at the start of the study, but we controlled the regressions for the fullness rating variable. A limitation of at-home VR studies is that it is difficult to collect precise data about the hardware and screen setup that participants used, since most people do not know this information. We did not know the screen size of each participant, but we pre-screened out those who did not have a laptop or a desktop and they could not proceed to the experiment if they did not maximize their screen. An important limitation was that the FPS was higher for the high- than for the low-fidelity VR, but both high- and low-fidelity had high FPS. The app was lightweight, so even the high-fidelity version could run in an old machine. This was pre-tested. Another technical consideration is that VR studies lack smell and touch, future studies should consider this using open-source devices for smell experiences which have been proposed as a solution [54].

In this study participants were given five minutes for the VR experience to standardize the exposure period between participants, which is a strength. However, some participants may have forgotten the foods while doing the questionnaire. Future studies should consider longer exposure periods or a back button to review the scene.

It is possible that the appearance of the food and environment from our study did not seem realistic for the participants, despite being created by a high-quality photogrammetry process. Future work may need to consider more accurate processes to capture lighting, lighting reflection, and coloring contrasts [55], and test models for higher fidelity of foods to test for the impact of realism on food choices. Moreover, additional studies are needed to test the individual effects of lighting, geometry and rendering quality separately. Another study could also test for different levels of food fidelity in the same stable environment to assess context. An immersive VR experiment, as opposed to a screen-based VR experiment such as this one, may also be needed to improve the realism and feeling of “being there”. It is crucial to understand to what extent virtual reality is perceived, in fact, as real by participants.

## Conclusion

Our results indicated that perceived realism, rather than visual fidelity, was associated with motivation, interest, and engagement in a food choice task. Overall, studies should measure and control for participants’ perception of realism in virtual and immersive virtual reality tasks. Additionally, manipulating visual fidelity appears to influence the selection of some foods, therefore caution should be used in studies that rely on specific food selections as part of the study outcomes. However, further studies are needed to determine why some foods may be affected by visual fidelity while others may not.

## Supporting information

S1 Table Probit regressions for the impact of visual fidelity and perceived realism on the number of times each food was chosen. (DOCX)

S2 Table Linear regressions for the impact of visual fidelity and perceived realism on the number of kilocalories chosen for each food separately. (DOCX)

S1 Data VisualFidelityOSF. (XLSX)
