# Supplementary material for: The impact of visual fidelity on screen-based virtual reality food choices: A randomized pilot study
Source: PLoS One. 2025 Jan 30;20(1):e0312772. doi: 10.1371/journal.pone.0312772 (PMC11781705; doi:10.1371/journal.pone.0312772)
Supplement: S1 Table — (DOCX) [file pone.0312772.s001.docx]

| **Supplementary Table 1**. Probit Regressions for the Impact of Visual Fidelity and Perceived Realism on the Number of Times Each Food Was Chosen | | | | | | | | | | | | |
| --- | --- | --- | --- | --- | --- | --- | --- | --- | --- | --- | --- | --- |
|  |  | Simple models | | | |  |  | Combined corrected models* | | | |  |
|  | Visual fidelity | | | Perceived realism | | | Visual fidelity | | | Perceived realism | | |
|  | B | SE | *p* | B | SE | *p* | B | SE | *p* | B | SE | *p* |
| **Low-energy dense foods (number)** | | | |  |  |  |  |  |  |  |  |  |
| Tomato | -0.150 | 0.28 | 0.60 | 0.008 | 0.01 | 0.32 | -0.190 | 0.31 | 0.54 | 0.008 | 0.01 | 0.23 |
| Corn | 0.040 | 0.29 | 0.89 | 0.014 | 0.01 | 0.10* | -0.036 | 0.32 | 0.91 | 0.014 | 0.01 | 0.11 |
| Strawberry | -0.048 | 0.28 | 0.86 | -0.010 | 0.01 | 0.20 | 0.065 | 0.30 | 0.83 | -0.011 | 0.01 | 0.17 |
| Raspberry | -0.242 | 0.29 | 0.41 | 0.005 | 0.01 | 0.49 | -0.397 | 0.33 | 0.23 | 0.006 | 0.01 | 0.50 |
| Cherry | 0.199 | 0.31 | 0.52 | 0.002 | 0.01 | 0.79 | 0.215 | 0.34 | 0.53 | 0.001 | 0.01 | 0.91 |
| Grapes | 0.040 | 0.29 | 0.89 | 0.002 | 0.01 | 0.76 | 0.188 | 0.32 | 0.55 | 0.001 | 0.01 | 0.87 |
| Banana | -0.210 | 0.28 | 0.45 | 0.006 | 0.01 | 0.41 | 0.011 | 0.31 | 0.97 | 0.006 | 0.01 | 0.43 |
| Pear | -0.243 | 0.32 | 0.45 | 0.036 | 0.01 | 0.01* | -0.290 | 0.42 | 0.49 | 0.035 | 0.01 | 0.012* |
| Apple | -0.129 | 0.31 | 0.68 | 0.004 | 0.01 | 0.59 | 0.275 | 0.36 | 0.44 | 0.003 | 0.01 | 0.70 |
| Kiwi | -0.059 | 0.32 | 0.86 | -0.001 | 0.01 | 0.88 | 0.129 | 0.36 | 0.72 | -0.001 | 0.01 | 0.90 |
| **High-energy dense foods (number)** | | | |  |  |  |  |  |  |  |  |  |
| Bread | -0.295 | 0.31 | 0.34 | -0.002 | 0.01 | 0.84 | -0.358 | 0.35 | 0.30 | 0.000 | 0.01 | 0.97 |
| Egg | -0.745 | 0.29 | 0.01* | -0.015 | 0.01 | 0.05* | -0.694 | 0.32 | 0.03* | -0.016 | 0.01 | 0.05* |
| Bacon | -0.533 | 0.30 | 0.07* | -0.010 | 0.01 | 0.18 | -0.428 | 0.32 | 0.18 | -0.013 | 0.01 | 0.10* |
| Cheese | -0.600 | 0.31 | 0.05* | 0.003 | 0.01 | 0.74 | -0.604 | 0.33 | 0.07* | 0.003 | 0.01 | 0.71 |
| Burger | -0.118 | 0.27 | 0.67 | -0.001 | 0.01 | 0.88 | -0.160 | 0.30 | 0.59 | 0.000 | 0.01 | 0.99 |
| Fries | -0.137 | 0.28 | 0.62 | -0.003 | 0.01 | 0.71 | -0.221 | 0.31 | 0.47 | -0.002 | 0.01 | 0.79 |
| Pizza | 0.112 | 0.11 | 0.69 | -0.003 | 0.01 | 0.70 | 0.014 | 0.31 | 0.96 | -0.002 | 0.01 | 0.81 |
| Cookie | 0.033 | 0.32 | 0.92 | -0.006 | 0.01 | 0.47 | -0.154 | 0.35 | 0.66 | -0.005 | 0.01 | 0.57 |
| Croissant | -1.000 | 0.35 | 0.005* | -0.009 | 0.01 | 0.27 | -1.367 | 0.42 | 0.001* | -0.006 | 0.01 | 0.55 |
| Cake | 0.052 | 0.28 | 0.85 | 0.002 | 0.01 | 0.76 | 0.077 | 0.30 | 0.80 | 0.003 | 0.01 | 0.69 |
| Muffin | -0.413 | 0.29 | 0.14 | -0.005 | 0.01 | 0.49 | -0.196 | 0.31 | 0.53 | -0.008 | 0.01 | 0.32 |
| SE is standard error. Significance is based on 95% CI. ^a^ Visual fidelity and perceived realism combined in a Probit regression adjusted for external food cue reactivity, fullness rating, and ethnicity. Visual fidelity was a dummy variable with high=1 and low=0. | | | | | | | | | | | | |
|  | | | | | | | | | | | | |
