# Supplementary material for: The impact of visual fidelity on screen-based virtual reality food choices: A randomized pilot study
Source: PLoS One. 2025 Jan 30;20(1):e0312772. doi: 10.1371/journal.pone.0312772 (PMC11781705; doi:10.1371/journal.pone.0312772)
Supplement: S2 Table — (DOCX) [file pone.0312772.s002.docx]

| **Supplementary Table 2.** Linear Regressions for the Impact of Visual Fidelity and Perceived Realism on the Number of Kilocalories Chosen for Each Food Separately | | | | | | | | | | | | |
| --- | --- | --- | --- | --- | --- | --- | --- | --- | --- | --- | --- | --- |
|  |  | Simple Models | | | |  |  | Combined corrected models^a^ | | | |  |
|  | Visual fidelity | | | Perceived realism | | | Visual fidelity | | | Perceived realism | | |
|  | B | SE | *p* | B | SE | *p* | B | SE | *p* | B | SE | *p* |
| **Low-energy dense foods (in kcal)** | | |  |  |  |  |  |  |  |  |  |  |
| Tomato | -1.210 | 2.31 | 0.60 | 0.060 | 0.06 | 0.33 | -1.589 | 2.50 | 0.53 | 0.065 | 0.06 | 0.30 |
| Corn | 1.538 | 11.28 | 0.89 | 0.473 | 0.29 | 0.11 | -1.482 | 12.26 | 0.90 | 0.482 | 0.31 | 0.12 |
| Strawberry | -0.653 | 3.86 | 0.87 | -0.127 | 0.10 | 0.22 | 0.988 | 4.22 | 0.82 | -0.140 | 0.11 | 0.19 |
| Raspberry | -4.656 | 5.67 | 0.41 | 0.106 | 0.15 | 0.49 | -6.654 | 6.14 | 0.28 | 0.084 | 0.15 | 0.59 |
| Cherry | 4.108 | 6.42 | 0.52 | 0.047 | 0.17 | 0.79 | 4.198 | 7.16 | 0.56 | 0.024 | 0.18 | 0.89 |
| Grapes | 1.171 | 8.58 | 0.89 | 0.068 | 0.23 | 0.79 | 5.443 | 9.44 | 0.57 | 0.025 | 0.24 | 0.92 |
| Banana | -7.671 | 10.34 | 0.46 | 0.225 | 0.27 | 0.41 | -0.222 | 11.18 | 0.98 | 0.223 | 0.28 | 0.43 |
| Pear | -3.967 | 5.31 | 0.46 | 0.397 | 0.14 | 0.004* | -4.002 | 5.46 | 0.47 | 0.394 | 0.14 | 0.005* |
| Apple | -2.284 | 5.52 | 0.68 | 0.081 | 0.18 | 0.58 | 3.613 | 5.83 | 0.54 | 0.048 | 0.15 | 0.74 |
| Kiwi | -0.980 | 5.41 | 0.86 | -0.022 | 0.14 | 0.88 | 2.568 | 5.91 | 0.67 | -0.020 | 0.15 | 0.89 |
| **High-energy dense foods (in kcal)** | | |  |  |  |  |  |  |  |  |  |  |
| Bread | -22.632 | 23.98 | 0.35 | -0.129 | 0.64 | 0.84 | -22.710 | 25.63 | 0.38 | -0.006 | 0.64 | 0.99 |
| Egg | -52.697 | 19.96 | 0.01* | -1.065 | 0.54 | 0.052* | -43.337 | 21.08 | 0.04* | -0.950 | 21.08 | 0.04* |
| Bacon | -82.822 | 45.80 | 0.07* | -1.645 | 1.23 | 0.19 | -60.405 | 49.72 | 0.23 | -1.860 | 1.24 | 0.14 |
| Cheese | -67.215 | 34.50 | 0.06* | 0.296 | 0.94 | 0.75 | -70.331 | 38.60 | 0.07* | 0.400 | 0.96 | 0.68 |
| Burger | -13.982 | 32.76 | 0.67 | -0.130 | 0.87 | 0.88 | -18.909 | 36.41 | 0.61 | 0.008 | 0.91 | 0.99 |
| Fries | -11.869 | 24.24 | 0.63 | -0.240 | 0.64 | 0.71 | -17.549 | 26.12 | 0.50 | -0.205 | 0.65 | 0.75 |
| Pizza | 11.836 | 29.49 | 0.69 | -0.299 | 0.78 | 0.70 | 1.862 | 32.55 | 0.96 | -0.188 | 0.81 | 0.89 |
| Cookie | 4.619 | 44.15 | 0.92 | -0.845 | 1.18 | 0.47 | -20.680 | 47.97 | 0.67 | -0.701 | 1.20 | 0.56 |
| Croissant | -102.479 | 34.21 | 0.004* | -1.056 | 0.95 | 0.27 | -135.981 | 36.26 | <0.001* | -0.711 | 0.90 | 0.43 |
| Cake | 7.964 | 42.63 | 0.85 | 0.343 | 1.13 | 0.76 | 11.542 | 46.98 | 0.807 | 0.457 | 1.17 | 0.70 |
| Muffin | -56.217 | 38.79 | 0.15 | -0.713 | 1.04 | 0.50 | -26.625 | 41.53 | 0.523 | -1.012 | 1.04 | 0.331 |
| SE is standard error. Significance is based on 95% CI.  ^a^Visual fidelity and perceived realism combined in a linear regression adjusted for fullness rating, external food cue reactivity, and ethnicity. Visual fidelity was a dummy variable with high=1 and low=0. | | | | | | | | | |  |  |  |
|  | | | | | | | | | | | | |
